# Exploring the effects of audiovisual incongruence on working memory performance in the combined 2-back+ Go/NoGo paradigm

**DOI:** 10.3389/fpsyg.2025.1578391

**Published:** 2025-06-04

**Authors:** Yang He, Tianqi Yang, Yuanbei Zhang, Kewei Sun, Qingjun Guo, Qiong Chen, Xuefeng Wang, Xiang Xu, Ping Wei, Shengjun Wu, Tao Xu

**Affiliations:** ^1^School of Psychology, Shanghai Normal University, Shanghai, China; ^2^Psychology Section, Secondary Sanatorium of Air Force Healthcare Center for Special Services, Hangzhou, China; ^3^Department of Military Medical Psychology, Air Force Medical University, Xi'an, China; ^4^Pilot Selection Bureau of PLA Air Force, Beijing, China; ^5^Air Force Bureau of Trainee Pilot Selection, Nanjing Central Division, Nanjing, China; ^6^Air Force Bureau of Trainee Pilot Selection, Jinan Central Division, Jinan, China

**Keywords:** audiovisual incongruence, working memory, cross-modal competition, cognitive load dynamics, 2-back+Go/NoGo, interference effect, sensory hierarchies

## Abstract

**Introduction:**

The human brain processes 83% of information visually and 11% auditorily, with visual perception dominating multisensory integration. While audiovisual congruence enhances cognitive performance, the impact of audiovisual incongruence on working memory (WM) remains controversial. This study investigated how audiovisual incongruence affects WM performance under varying cognitive loads.

**Methods:**

Two experiments employed a dual 2-back+Go/NoGo paradigm with 120 college students. Experiment 1 used alphanumeric stimuli (numbers/letters), while Experiment 2 utilized complex picture stimuli. Participants completed WM tasks under three conditions: visual-only, auditory-only, and incongruent audiovisual. Performance comparisons between unimodal and cross-modal conditions were analyzed using paired-samples t-tests.

**Results:**

Experiment 1 revealed visual interference on auditory WM (p <.05) but minimal auditory interference on visual WM. Experiment 2 demonstrated bidirectional interference between modalities (both *p* <.001), with cross-modal competition intensifying under high cognitive load. Results indicated interference patterns were mediated by cognitive load dynamics rather than fixed sensory hierarchies.

**Discussion:**

Audiovisual incongruence systematically disrupts WM performance, challenging conventional sensory dominance models. While low cognitive load permits strategic visual prioritization, high load triggers competitive cross-modal interactions. These findings suggest adaptive resource allocation mechanisms in WM that supersede strict visual supremacy principles, highlighting the context-dependent nature of multisensory integration.

## 1 Introduction

Cognitive science has long been a hotspot of research in the scientific community. Working memory (WM), the core of cognitive activity, is defined as a memory processing system with limited capacity for temporarily storing and managing information during the execution of cognitive tasks (Baddeley, 1992). In previous WM research, Baddeley proposed a WM model consisting of three components: a central executive system [containing refresh, switch and inhibit functions (Friedman and Miyake, 2017)], a verbal storage system (phonological loop) and a visual storage system (visuospatial sketchpad) (Baddeley, 2003). Later, a situational buffer capable of storing information from multiple templates that may come from multiple modalities (e.g., visual vs. auditory) was proposed. Working memory plays a very important role in higher cognitive functions such as speech, planning, reasoning, decision making, learning, thinking, problem solving, and spatial processing; it provides the transient storage and processing necessary for performing higher cognitive tasks (Baddeley, 2012). Therefore, WM has become the focus of attention in the field of cognitive science. The N-back and Go/NoGo tasks are classic research paradigms used to investigate WM (Diamond, 2013; Jaeggi et al., 2010). In the N-back task, subjects are asked to judge whether the currently presented stimulus is the same as the stimulus presented N-1 stimuli ago. The 2-back task is the most representative and is widely used by researchers (Breitling-Ziegler et al., 2020). In the Go/NoGo task, two different letters, such as the letter “O” and the letter “X,” are randomly alternated, and subjects are asked to respond to the letter “O” (the so-called Go stimulus) while restraining from responding to the letter “X” (the so-called NoGo stimulus). Erroneous responses to NoGo stimuli are often considered a measure of the difficulty of inhibiting responses in WM (Diamond, 2013).

Similarly, research on audiovisual interaction, as another indispensable component of cognitive activity, has been accumulating recently, and it has become a hot research topic in the field of cognitive science in the past decade. Audiovisual interaction refers to the fact that when the visual and auditory channels acquire information about the same thing, there is an overlap of information from different senses. Audiovisual interaction refers to the phenomenon where, when the visual and auditory channels receive information about the same object, there is an overlap of sensory information from these two modalities. This overlapping information undergoes interactive processes during brain integration, leading to distinct cognitive consequences: it enhances performance (e.g., faster reaction times, improved detection accuracy) under congruent conditions, whereas it disrupts processing (e.g., response errors, prolonged latency) under incongruent conditions—a distinction critical for understanding multisensory perception (Tai et al., 2022; Bulkin and Groh, 2006). Delving deeper, this interaction encompasses both the behavioral effects of audiovisual (in)congruence and the neurocognitive mechanisms underlying crossmodal integration. For such interaction to occur, three prerequisites must be satisfied. First, spatially coincident signals are parsed as originating from the same object, a principle rooted in the ventriloquist effect (Bertelson et al., 1994). Second, temporally overlapping signals are bound to the same source, as demonstrated by the sound-induced flash illusion (Shams et al., 2000). Third, empirically, signals historically associated with the same object (e.g., a dog’s bark and appearance) are perseveratively linked, even in the absence of current sensory congruence—a phenomenon highlighting predictive coding in multisensory processing (Bulkin and Groh, 2006). In this way, due to the dominant role of a single modality, when information originating from the same object overlaps in congruent or incongruent conditions, cognition is correspondingly facilitated or interfered with, producing differential results (Tai et al., 2022). However, in recent years, research has not only focused on the audiovisual interaction itself but also extended to the effects of other factors (e.g., training, cognitive load, and brain mechanisms (Csonka et al., 2021)) on the process of audiovisual integration, which provides a basis for more in-depth study of theories of information processing during audiovisual interaction.

Early research on the effects of audiovisual interaction on cognitive function suggested that when audiovisual information is congruent, it generally facilitates audiovisual interaction. For example, in the classic sound-induced flash illusion effect, when visual flashes are accompanied by an unequal number of auditory sounds presented sequentially or simultaneously within 100 ms, individuals perceive that the number of visual flashes is equal to the number of auditory sounds (Abadi and Murphy, 2014; Cecere et al., 2015). Andersen et al. (2004) and Mishra et al. (2007) even demonstrated that when audiovisual stimuli are congruent (i.e., one flash accompanied by one pure tone or two flashes accompanied by two pure tones), subjects not only showed an increase in response accuracy but also a decrease in reaction time. Botta et al. (2011) investigated how spatial attention driven by single-modality (visual/auditory) cues and multimodal (audiovisual) cues biased information in visuospatial WM and found that compared to visual-only cues, spatially congruent multimodal cues were more efficient in visuospatial WM, showing greater attentional effects. Patching and Quinlan (2002) studied the relationship between the vertical position of visual stimuli and the auditory frequency, reporting that the speed of subjects’ categorization of visual stimuli was significantly increased when there was a consistent auditory stimulus present.

In addition, audiovisual interactions have been reported in the mutual reinforcement between auditory and visual information from the same object or spatial location, i.e., when an object presents multimodal information, such as visual and auditory information, paying attention to one of these modalities promotes perception in the other modality. For example, Busse et al. (2005) found that when attention was drawn to the visual aspects of an object, the brain’s processing of auditory information about the object was enhanced, even if the auditory information was task-irrelevant. McDonald et al. (2000) found that when attention was drawn to auditory aspects, judgments of visual information appearing at the same spatial location were also enhanced. These studies suggest that when considering the same object or the same spatial location, attention in either the visual or auditory modality will “spread” to facilitate cognition in the other modality. The main reason is that when the two sensory systems, i.e., visual and auditory, receive information from the same object, the combination of different signals can improve the accuracy and speed of cognitive results (Tomko and Proctor, 2017). For example, in daily interactions, if you can see another person’s lips as they talk, you can better understand what the other person wants to express. Communication that is only heard and not seen tends to be less effective than face-to-face conversation.

However, visual and auditory information are not necessarily congruent, and when the two deviate slightly, it is unclear whether audiovisual interaction facilitates or interferes with cognitive outcomes. Early studies found that when visual and auditory information are encoded differently and attention to different stimuli in both modalities is needed simultaneous, clear competition will occur between the two modalities. For example, in the classic McGurk effect, discovered by McGurk and MacDonald (1976) in 1976, a person who appears to say “ga” in a video recording paired with the sound “ba” is heard to say “da” by subjects. That is, when a subject sees a mismatch between the mouth shape (visual stimulus) of articulation and the auditory stimulus, the subject is influenced by the visual stimulus and misperceives the sound (McGurk and MacDonald, 1976). Further subsequent studies by Evans and Treisman (2010) have shown that when visual or auditory stimuli with different encoded information are used as the target stimuli and inconsistent auditory or visual distractor stimuli are added, the brain responses of subjects are inhibited to some extent, as well as the possibility that this interference may be related to working memory load.

WM theory is a basic theory of cognitive processing load (Sun and Liu, 2013). Specifically, WM, as a cognitive processing resource, has a limited capacity, and when more cognitive resources are needed to process stimuli, the cognitive processing load on WM is greater (Xu et al., 2023). Liu et al. (2021) showed that the experimental task itself is a major factor influencing cognitive load, and the cognitive burdens of the task affect both WM accuracy (Luck and Vogel, 1997) and interference due to external distractors (Zhang et al., 2011). Therefore, the type of stimuli in the physical environment inevitably affects cognitive load. Second, the most important factors affecting cognitive load are the essential features of the stimulus material (nature and source), the organization and presentation of the material, and so on. The nature of stimulus materials inevitably influences the cognitive load of learners (Liu and Sun, 2016). Furthermore, recent research has shown that people typically have more difficulty remembering faces (Wu and Huang, 2018), pictures (Schmeck et al., 2015) and spatial locations (Peynircioǧlu et al., 2017) than simple numbers and symbols. This difficulty arises because complex stimuli, such as pictures, impose greater demands on WM due to their detailed visual features and the need for deeper semantic processing. For instance, processing intricate visual scenes requires continuous integration of multiple elements (e.g., shapes, spatial relationships, and contextual details), which consumes more cognitive resources compared to simple symbols like numbers or letters (Konstantinou and Lavie, 2013; Neokleous et al., 2016). The main reason is that the human cognitive processing load is limited by WM, which is positively correlated with the allocation of cognitive resources in the human brain (Horat et al., 2016). When cognitive resources are allocated and managed in a targeted manner, the cognitive load is negatively correlated with cognitive resource reserves, i.e., the lower the cognitive load perceived by an individual, the more adequate the cognitive resource reserves and the easier it is to suppress irrelevant interfering stimuli (Wickens et al., 1983).

Conversely, some researchers hold the opposite view and suggest that there may be synergistic cooperation between incongruent visual and auditory information in the process of cognitive resource allocation. For example, Chen and Yeh (2009) and Van Der Burg et al. (2008) found that the addition of either congruent or incongruent auditory stimuli enhanced the perception of visual target stimuli, while Odgaard et al. (2004) found that the addition of visual stimuli while presenting white noise helped to accelerate the perception of auditory white noise. Stefanics et al. (2005) investigated cross-modal interactions by presenting visual targets with incongruent auditory or tactile stimuli in a visual selection task. Their results demonstrated that audiovisual stimuli elicited significantly shorter reaction times compared to unimodal visual conditions, with concurrent reductions in P300 latency observed in event-related potentials. This behavioral-electrophysiological dissociation indicates that cross-modal inputs accelerate perceptual processing even under stimulus incongruence (Andres et al., 2011; Ganesh et al., 2014; Senkowski et al., 2011). Notably, the facilitation effect was stronger for audiovisual than visuotactile pairings, highlighting modality-specific advantages in multisensory integration (Stefanics et al., 2005).

Interestingly, we found that the visual and auditory stimuli in the above studies primarily manipulated incongruence through mismatched perceptual attributes (e.g., conflicting spatial locations or temporal frequencies), which represents a critical theoretical limitation given that real-world cognitive conflicts often originate from competing behavioral goals rather than sensory discrepancies. Specifically, these paradigms introduced auditory stimuli as secondary inputs to a primary visual task, such as visual target detection accompanied by concurrent task-irrelevant tones. This approach inadvertently conflated perceptual incongruence with task hierarchy effects, potentially obscuring the true nature of cross - modal interactions. To address this gap, our paradigm establishes executive - level incongruence through orthogonal task sets: the visual task is a 2-back working memory updating task, requiring continuous maintenance and matching of sequential spatial information, while the auditory task is a Go/NoGo task demanding the suppression of prepotent motor responses to low - frequency tones. This design creates a novel form of cross-modal conflict where competing cognitive operations—information updating in the visual domain versus response suppression in the auditory domain—tax the central executive’s conflict monitoring resources (Norman and Shallice, 1986), rather than relying on simple perceptual feature mismatches. Paradoxically, such executive conflict enhances multisensory integration efficiency, as electrophysiological studies have shown that competition for prefrontal resources during this cross-modal conflict boosts gamma-band synchronization between frontoparietal regions and sensory cortices (Senkowski et al., 2007). Moreover, EEG evidence indicates increased cross-modal phase coherence under high cognitive load (Talsma et al., 2006), suggesting that despite the stimulus incongruence, the brain can enable synergistic processing, likely due to its adaptive mechanisms that optimize the allocation of cognitive resources to integrate information from different modalities even when faced with conflicting task demands.

The dominance of unimodal visual processing in such contexts remains debated. Welch et al. (1986) suggested that the establishment of dominance is related mainly to the sensitivity of modalities and that more sensitive modalities are prone to dominate the processing of bimodal stimuli. Furthermore, approximately 83% of the information people receive comes from the visual channel while only 11% comes from the auditory channel (Feng and Yang, 2018); thus, visual perception is prioritized in the integration of sensory input. Second, from the perspective of cognitive resource allocation, visual attention is essentially a resource allocation scheme that arises under the constraints of multiple factors and can redistribute the limited information processing capacity of humans (Kahneman and Tversky, 1973). Therefore, when a stimulus in a second modality is added, cognitive resources are, reallocated and the visual modality is prioritized, i.e., the synergistic compensation of visual dominance over auditory dominance is achieved when the audiovisual stimuli are incongruent. While visual dominance is well-documented in multisensory integration (Colavita, 1974), this phenomenon is context-dependent rather than absolute. The sound-induced flash illusion (Shams et al., 2000) demonstrates auditory dominance in temporal perception, while Fox et al. (2009) show attention can be flexibly directed toward auditory stimuli in dual-task contexts. These contradictions highlight the need to dissociate perceptual hierarchies from domain-general cognitive load effects—a gap our study directly addresses.

WM, which is central to human cognitive function, plays a pivotal role in higher-order cognition. Visual and auditory processing are subordinate to the integration and regulation of information from the two modalities when making judgments at the consciousness and behavioral levels. Recently, many important results have been achieved in cognitive research on vision and hearing alone. However, some key questions remain to be addressed in the study of audiovisual integration and cognition in terms of information processing and processing methods, such as: (1) whether audiovisual incongruence primarily impairs or facilitates cognitive performance, and (2) whether the direction and magnitude of such interference are modulated by domain-general cognitive load rather than fixed sensory hierarchies. To address these questions, we propose three hypotheses anchored in resource competition frameworks: (H1) Audiovisual incongruence will impair WM performance relative to unimodal conditions, reflecting interference from competing task-set configurations (Kornblum et al., 1990); (H2) Under low cognitive load, interference will be task-contingent and asymmetric—visual WM updating demands (2-back) will dominate resource allocation, exerting stronger cross-modal interference on auditory inhibition (Go/NoGo) than vice versa (Lavie et al., 2004); (H3) High visual cognitive load will induce bidirectional interference by depleting global attentional resources, overriding modality-specific competition patterns (Souza et al., 2018). Experiments 1 and 2 test these hypotheses: Experiment 1 evaluates H1 and H2 using simple alphanumeric stimuli (low visual load), while Experiment 2 examines H3 with complex pictorial stimuli under high visual load. The use of complex pictorial stimuli in Experiment 2 is particularly effective in manipulating cognitive load because, as mentioned earlier, complex stimuli demand more cognitive resources for processing. This increased resource demand leads to a higher cognitive load, which allows us to investigate how audiovisual incongruence interacts with cognitive load in a more pronounced way.

In summary, the present study adopts a novel operationalization of audiovisual incongruence based on response conflict rather than stimulus attribute mismatch, grounded in the classic combined 2-back (visual) + Go/NoGo (auditory) dual-task paradigm. Following Kornblum’s dimensional overlap theory (Kornblum et al., 1990), we define incongruence as a state where concurrent audiovisual stimuli demand mutually incompatible cognitive operations. Specifically, the visual task (2-back) requires continuous updating and matching of sequential visual information, while the auditory task (Go/NoGo) requires inhibition of prepotent responses to low-frequency tones. Critical incongruence arises when the visual WM updating process (e.g., judging “whether the current number matches the one two steps back”) competes with the auditory response inhibition process (e.g., “withholding keypress to low-pitch tones”). This creates cross-modal executive conflict distinct from traditional stimulus-level incongruence (e.g., McGurk effect). Thus, this study provides a theoretical framework for understanding how audiovisual incongruence disrupts WM-based information processing.

## 2 Experiment 1: effects of audiovisual incongruence based on numbers or letters on the performance on a WM task

### 2.1 Methods

#### 2.1.1 Participants

Referring to previous studies, the sample size was estimated using G*Power 3.1 software (Faul et al., 2009) with the following parameters: *f* = 0.25, *α* = 0.05, and 1 - *β* = 0.80. A minimum total sample size of 54 was calculated. Therefore, 60 undergraduate cadets from a military school, aged 20.27 ± 0.92 years, were openly recruited to participate in this study using random sampling. All subjects were male, right-handed, with normal vision and hearing, no color blindness or color deficiency, and normal intelligence. All cadets voluntarily participated in the experiment, signed an informed consent form and received a monetary reward for participating at the end of the experiment. The study was conducted in accordance with the Declaration of Helsinki and approved by the Ethical Review Committee of Xijing Hospital (KY20224106-1).

#### 2.1.2 Stimuli

The visual stimuli were 8 numbers (i.e., 3, 6, 12, 15, 21, 24, 30, and 33) and 4 letters (W, N, E, and S), for a total of 12 stimuli. The auditory stimuli had a loudness of 70 dB and were categorized into 2 types: low-frequency tones (262 Hz) and high-frequency tones (524 Hz).

#### 2.1.3 Experimental design

The present study employed a one-factor, three-level within-subjects design, encompassing three experimental tasks: (1) a visual-only WM task, requiring participants to memorize numbers or letters; (2) an auditory-only WM task, involving judgments about pure-tone stimuli (low or high frequency); and (3) an audiovisual WM dual task, integrating both modalities. Critically, to distinguish procedural incongruence from perceptual conflict, the stimuli utilized in both modalities were perceptually neutral (numbers/letters for vision, pure tones for audition). In the dual-task condition, participants concurrently executed the following tasks: the Visual 2-back, where they determined if the current number/letter matched the one shown two steps back, necessitating WM updating, and the Auditory Go/NoGo, where they responded to high-frequency tones (Go) while refraining from responding to low-frequency tones (NoGo), requiring inhibition.

Incongruence in this paradigm stemmed solely from the competition between concurrent cognitive operations (i.e., updating versus inhibiting), rather than from mismatched stimulus characteristics. This design ensures that any observed interference originates from domain-general resource competition (such as attentional control) rather than stimulus-level incompatibility. To mitigate potential order effects, six task permutation sequences were utilized (see Table 1), with 10 participants assigned to each sequence. By disentangling perceptual conflict from task demands, our approach aligns with Kornblum et al.’s (1990) dimensional overlap theory, which posits that conflict arises from overlapping processing pathways, not from stimulus incongruity.

**Table 1:** Six permutation sequences for the three experimental task conditions.

Sequence	Combination of task types
1	Visual → auditory → audiovisual
2	Visual → audiovisual → auditory
3	Auditory → visual → audiovisual
4	Auditory → audiovisual → visual
5	Audiovisual → visual → auditory
6	Audiovisual → auditory → visual

“Visual” represents a visual-only working memory task test, “auditory” represents an auditory-only working memory task, and “audiovisual” represents the audiovisual working memory dual task.

#### 2.1.4 Experimental procedure

##### 2.1.4.1 Visual-only working memory task

**Figure 1:**
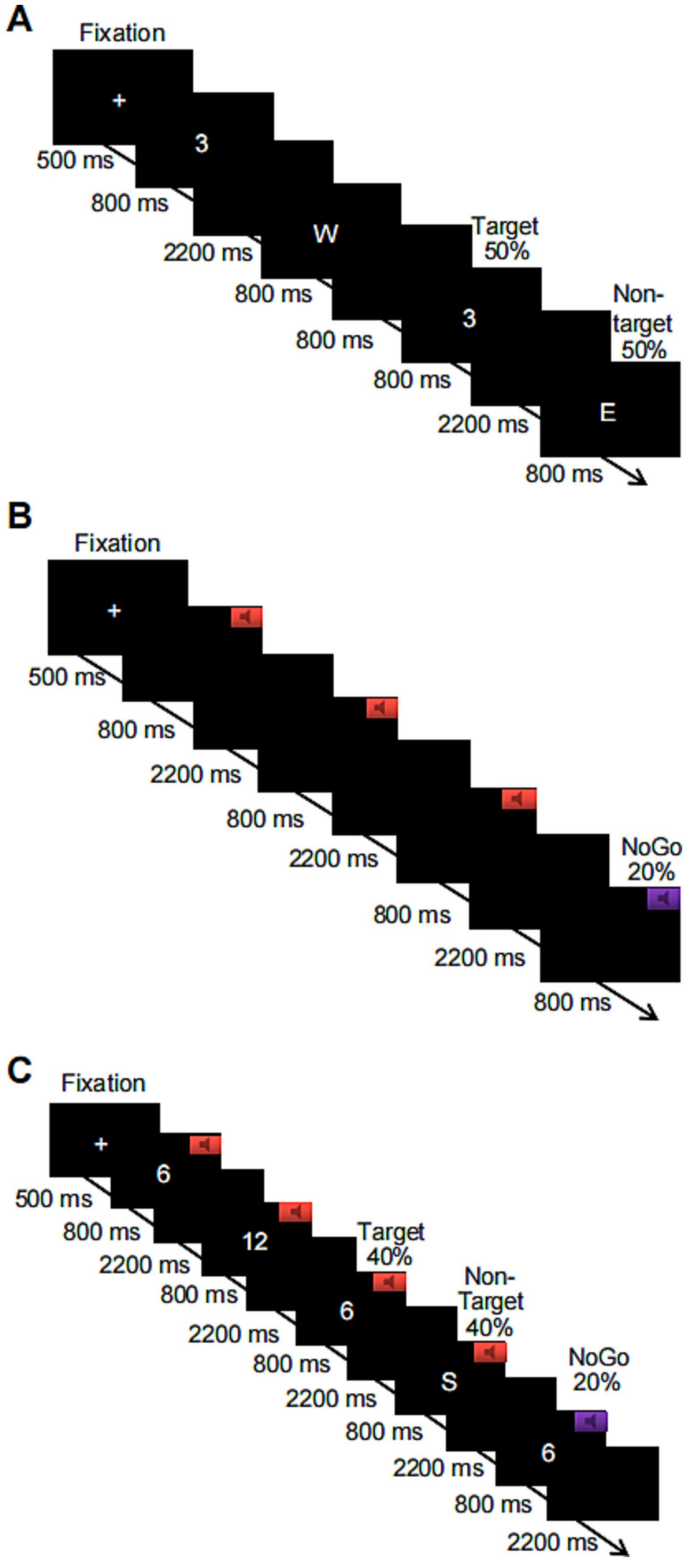
Schematic flowchart of three levels of the working memory task based on numbers or letters, where **(A)** shows the visual-only 2-back task, **(B)** shows the auditory-only Go/NoGo task, and **(C)** shows the combined visual 2-back + auditory Go/NoGo dual task.

The 2-back paradigm was used to assess subjects’ performance on a visual WM task. Before the start of the task, a fixation cross (“+”) appeared in the center of the screen for 500 ms, reminding subjects to focus on the task. When the “+” disappeared, a number or letter appeared randomly on the computer screen. Each stimulus lasted 800 ms, and the next stimulus was automatically presented after the participant pressed a key in response or after 3,000 ms. The target stimulus and the nontarget stimulus each appeared 50% of the time, and subjects were required to judge whether the number or letter appearing on the current screen was the same as the number or letter presented previously according to the instructions. If the stimuli were the same, participants answered by pressing the “F” key on the keyboard, and if the stimuli were not the same, participants answered by pressing the “J” key on the keyboard. The task consisted of 1 block with a total of 80 trials. Additionally, there was a 1-min (20-trial) practice phase before the formal experimental task, in which participants received feedback for correct and incorrect responses. When the level of accuracy in the practice phase reached 80%, subjects were assumed to understand the task; otherwise, the subject had to repeat the practice. The duration of the entire experimental task was approximately 5 min, and the flowchart is shown in Figure 1A. The outputs were subject response accuracy and reaction time for target and nontarget stimuli.

##### 2.1.4.2 Auditory-only working memory task

The Go/NoGo paradigm was used to assess performance on an auditory WM task. Before the start of this task, a “+” appeared in the center of the screen for 500 ms, reminding subjects to focus on the task. When the “+” disappeared, the subject heard two randomly alternating acoustic stimuli, each of which lasted for 800 ms. The next stimulus was automatically presented after the subject pressed a key in response or after 3,000 ms. If the sound stimulus was a high-frequency “beep” (524 Hz), i.e., the Go stimulus, the subject was asked to press the “space” key on the keyboard to respond; if the sound stimulus was a low-frequency “beep” (262 Hz), i.e., the NoGo stimulus, subjects restrained their response and did not press the key. These NoGo trials accounted for 20% of all experimental trials. The task consisted of 1 block with a total of 100 trials. A 1-min (20-trial) practice phase preceded the start of the formal experimental task, in which participants received feedback on correct or incorrect responses. When the level of accuracy in the practice phase reached 80%, subjects were assumed to understand the task; otherwise, the subject had to repeat the practice. The duration of the entire experimental task was approximately 6 min; a flowchart is shown in Figure 1B. The output was subject response accuracy on the NoGo trials and response time on the Go trials.

##### 2.1.4.3 The audiovisual working memory dual task

A combination of the 2-back task + Go/NoGo task was used to assess performance on a WM task under audiovisual incongruence. Before the start of the task, a “+” appeared in the center of the screen for 500 ms to remind subjects to focus on the task. When the “+” disappeared, a number or a letter was randomly presented on the computer screen, and one of the two sound stimuli was randomly played through the headphones. The duration of the stimulus pair (sound and number or sound and letter) was 800 ms, and the next stimulus was automatically presented after the participant pressed a key or after 3,000 ms. If the sound stimulus was a high-frequency tone, the subject was asked to determine whether the number or letter appearing on the current screen was the same as the number or letter that was one away from it. If the stimuli were the same, the “F” key on the keyboard was pressed; if the stimuli were not the same, the “J” key on the keyboard was pressed. If the sound stimulus was a low-frequency tone, subjects were not supposed to press a key to respond, regardless of whether the number or letter appearing on the current screen was the same as or different from the number or letter that appeared previously. These trials with restrained responses accounted for 20% of the total number of trials in the entire experiment. The task contained 1 block with a total of 100 trials. The combined visual 2-back + auditory Go/NoGo dual task had the same number of target trials as the visual-only 2-back task and the auditory-only Go/NoGo task to facilitate subsequent comparisons. Additionally, a 1-min (20-trial) practice phase was provided before the start of the formal experimental task in both cases, and feedback regarding correct and incorrect responses was provided. When the level of accuracy in the practice phase reached 80%, subjects were assumed to understand the task and were entered in the formal experiment; otherwise, the subject had to repeat the practice. The duration of the entire experimental task was approximately 6 min, and a flowchart of the task is shown in Figure 1C. The outputs were subject response accuracy and response time on the target and nontarget stimuli in the 2-back task condition and the response accuracy and response time in the NoGo trials of the Go/NoGo task.

#### 2.1.5 Data processing

Data were organized and statistically analyzed using Excel 2019 and SPSS 25.0. First, the data were screened, and datapoints more than 3 standard deviations from the mean were excluded. This resulted in the exclusion of data from one subject; all the data of the remaining 59 individuals were included in the later statistical analysis. In addition, the behavioral data were analyzed using the statistical software SPSS 25.0, with paired-samples *t* tests used to compare the 59 subjects’ accuracy and reaction times between the visual-only 2-back task and the audiovisual dual task, as well between the auditory-only Go/NoGo task and the audiovisual dual task. Finally, the interference effect of the audiovisual interaction on WM task performance was examined using the effect size r_pb_^2^ = t^2^/(t^2^ + df), where the effect size is considered small for 0.010 ≤ r_pb_^2^ < 0.059, medium for 0.059 ≤ r_pb_^2^ < 0.138, and large for r_pb_^2^ ≥ 0.138 (Quan, 2003). For the above statistical analyses, the significance threshold was set to *α* = 0.05.

### 2.2 Results

#### 2.2.1 Comparison of the auditory-only Go/NoGo task and the combined auditory Go/NoGo task + visual 2-back dual task

The accuracy and reaction time results were compared between the auditory-only Go/NoGo task and the auditory-only Go/NoGo condition in the audiovisual dual task using the visual 2-back stimuli as the interference stimuli, as shown in Table 2.

**Table 2:** Go/NoGo parameters in the single and dual tasks (mean ± standard deviation).

Task type	Memory condition	Accuracy	Reaction time (ms)
Single (auditory-only)	Go/NoGo	0.97 ± 0.05	510.08 ± 139.63
Dual (simultaneous audiovisual presentation)	Go/NoGo+2-back	0.95 ± 0.04	606.45 ± 129.05

**Figure 2:**
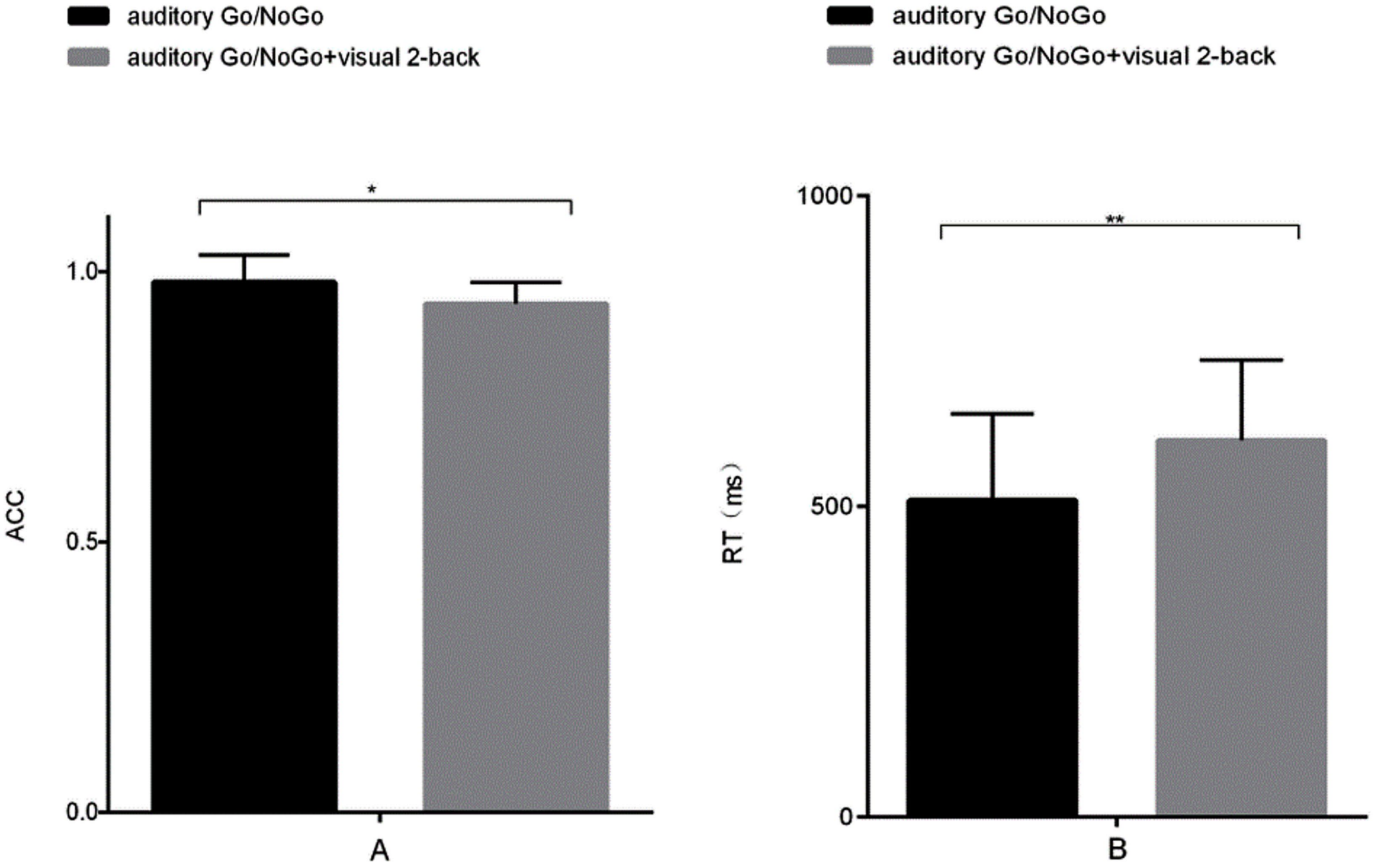
Accuracy and reaction time in the auditory-only Go/NoGo task versus the audiovisual dual task. **(A)** single-task (auditory) and dual-task (audiovisual) conditions for accuracy (ACC); **(B)** single-task (auditory) and dual-task (audiovisual) conditions for reaction time (RT). Note: ACC denotes response accuracy, RT denotes reaction time, * denotes *p* < 0.05, and ** denotes *p* < 0.01.

The accuracy of the auditory Go/NoGo condition in the audiovisual dual task was significantly lower than that in the auditory-only Go/NoGo task [*t* (57) = 2.044, *p* = 0.038, r_pb_^2^ = 0.067] (see Figure 2A). In terms of reaction time, the reaction time of the auditory Go/NoGo condition of the audiovisual dual task was significantly longer than that of the auditory-only Go/NoGo task [*t* (57) = −2.849, *p* = 0.004, r_pb_^2^ = 0.122] (see Figure 2B). In addition, in the combined auditory Go/NoGo + visual 2-back dual task, we calculated the correlation between accuracy and reaction time to determine whether there was a speed-accuracy trade-off, and the results showed no significant negative correlation between the two [r (57) = − 0.213, *p* > 0.05].

#### 2.2.2 Comparison of a visual-only 2-back task with the combined visual 2-back task + auditory Go/NoGo dual task

Using the auditory Go/NoGo task as a distractor stimulus, the accuracy and reaction time results were compared between the visual-only 2-back task and the visual 2-back condition in the audiovisual dual task, as shown in Table 3.

**Table 3:** 2-back parameters in single and dual tasks (mean ± standard deviation).

Task type	Memory condition	Accuracy	Reaction time (ms)
Single (visual-only)	2-back	0.94 ± 0.05	922.93 ± 179.22
Dual (simultaneous audiovisual presentation)	2-back	0.92 ± 0.04	1015.38 ± 177.54

**Figure 3:**
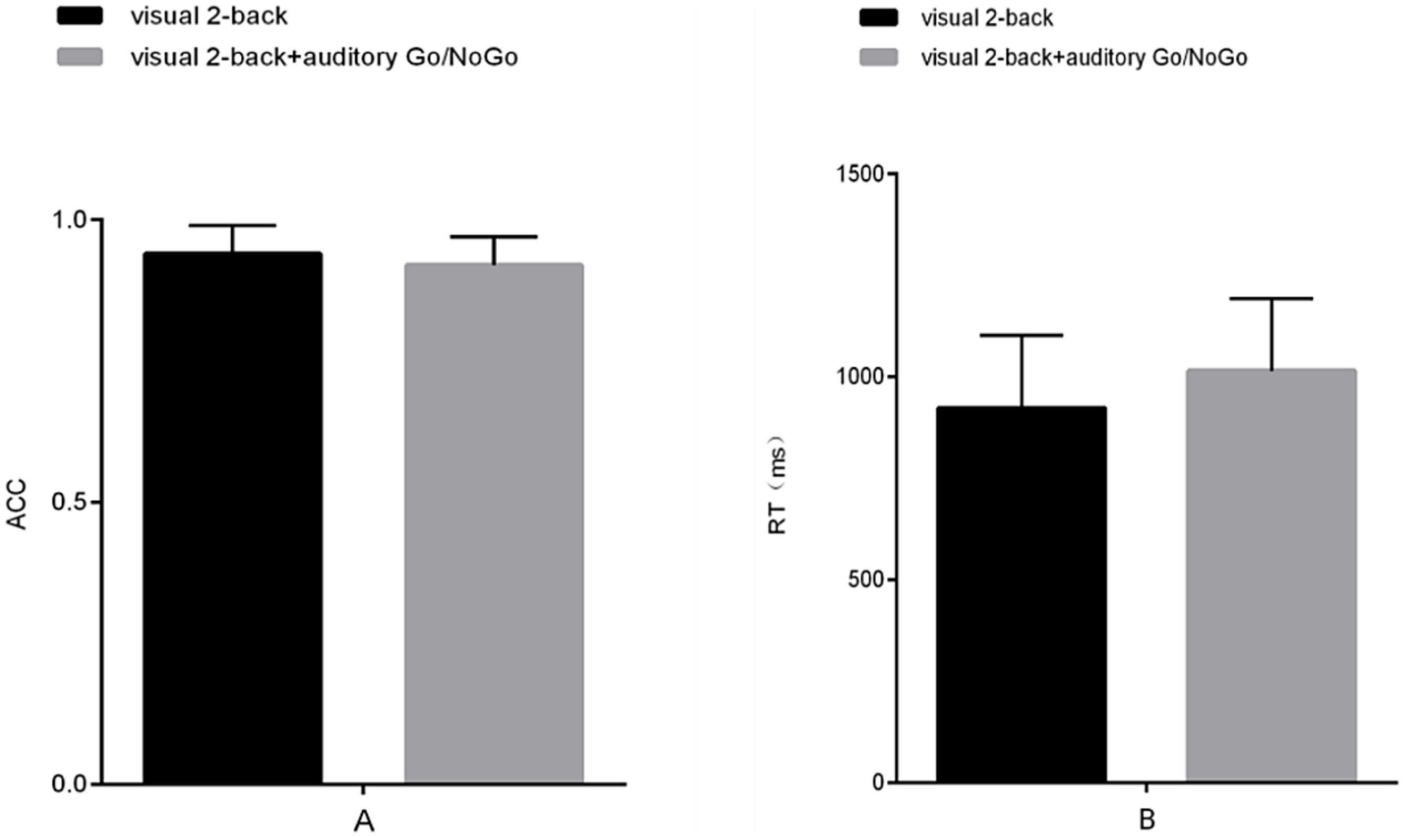
Accuracy and reaction time in the visual-only 2-back task versus the audiovisual dual task. **(A)** single-task (visual) and dual-task (audiovisual) conditions for accuracy (ACC); **(B)** single-task (visual) and dual-task (audiovisual) conditions for reaction time (RT). Note: ACC denotes response accuracy, and RT denotes reaction time.

The accuracy of the visual 2-back condition in the audiovisual dual task was lower than that in the visual-only 2-back task, but there was no significant difference between the two [*t* (57) = 1.748, *p* = 0.139, r_pb_^2^ = 0.050] (see Figure 3A). In terms of reaction time, the reaction time of the visual 2-back condition in the audiovisual dual task was longer than that of the visual-only 2-back task, but this difference only approached marginal statistical significance [*t* (57) = −1.858, *p* = 0.053, r_pb_^2^ = 0.056] (see Figure 3B). In addition, in the combined visual 2-back + auditory Go/NoGo dual task, we calculated the correlation between accuracy and reaction time to determine whether there was a speed-accuracy trade-off, and the results showed a significant negative correlation [r (57) = −0.457, *p* = 0.016].

### 2.3 Summary

The findings from the dual - task paradigm involving audiovisual WM based on numbers or letters indicate the potential presence of unidirectional interference from the visual to the auditory modality when processing information from audiovisual incongruence stimuli. Specifically, the visual modality appears to disrupt the completion of the auditory WM task performance, while the auditory modality exerts minimal interference on the visual WM task performance.

## 3 Experiment 2: a picture-based exploration of the effects of audiovisual incongruence on working memory task performance

Experiment 1 revealed that under conditions of low visual cognitive load, which were induced by simple alphanumeric stimuli, audiovisual incongruence gave rise to unidirectional interference. This interference was predominantly characterized by the dominance of visual WM updating (as assessed by the 2 - back task) over auditory inhibition (as measured by the Go/NoGo task), a finding that was consistent with Hypotheses H1 and H2. However, real-world cognitive scenarios frequently unfold under high-load conditions. For instance, pilots must process instrument panels while simultaneously responding to alarms. This highlights the necessity of investigating how cognitive load dynamics, rather than perceptual hierarchies, regulate cross-modal interference.

To address this gap, Experiment 2 replaces alphanumeric stimuli with complex pictorial stimuli (flight instrument panels). These stimuli demand higher-order visual processing (object recognition, spatial integration; Schmeck et al., 2015), validated in pilot studies to impose higher subjective complexity and slower reaction times than alphanumeric stimuli. Retaining the dual-task structure (2-back + Go/NoGo), this design isolates the role of cognitive load in modulating interference patterns (H3). If high visual load depletes domain-general resources, bidirectional interference should emerge as both modalities compete for residual capacity (Souza et al., 2018). This approach advances prior work by decoupling executive conflict (updating vs. inhibition) from perceptual incongruence, offering ecologically valid insights into adaptive multisensory integration.

### 3.1 Methods

#### 3.1.1 Participants

Referring to previous studies, the sample size was estimated using G*Power 3.1 software (Karr et al., 2018) with the following parameters: *f* = 0.25, *α* = 0.05, and 1 - *β* = 0.80. A minimum sample size of 54 subjects was calculated. There was no overlap of subjects between Experiment 1 and Experiment 2. Therefore, 60 undergraduate cadets of a military school, aged 19.75 ± 1.04 years, were openly recruited to participate in this study using random sampling. All subjects were male, right-handed, with normal vision and hearing, no color blindness or color weakness, and normal intelligence. Table 4 summarizes the sample characteristics and shows that there was no statistically significant difference in age between subjects in Experiment 1 and Experiment 2 (*p* > 0.05). In addition, all subjects voluntarily participated in the experiment and signed an informed consent form, and we paid them for participating after the experiment was completed.

**Table 4:** Demographic data of subjects in Experiment 1 and Experiment 2 (mean ± standard deviation).

Variable	Experiment 1	Experiment 1	*t*	*p*	r_pb_^2^
Number (*n*)	60	60	-	-	-
Age (years)	20.27 ± 0.92	19.75 ± 1.04	1.331	0.193	0.01

#### 3.1.2 Stimuli

**Figure 4:**
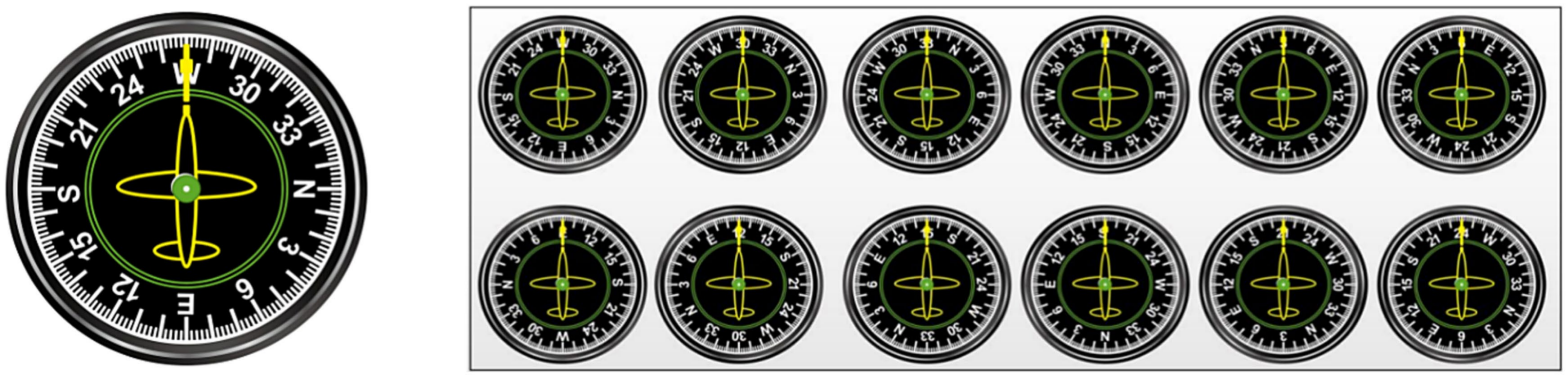
Twelve flight instrument panel pictures.

The visual stimuli were 12 pictures of flight instruments (see Figure 4). The auditory stimuli had a loudness of 70 dB and were categorized into 2 types: low-frequency tones (262 Hz) and high-frequency tones (524 Hz).

#### 3.1.3 Experimental design

Same as Experiment 1.

#### 3.1.4 Experimental procedure

##### 3.1.4.1 Visual-only working memory task

**Figure 5:**
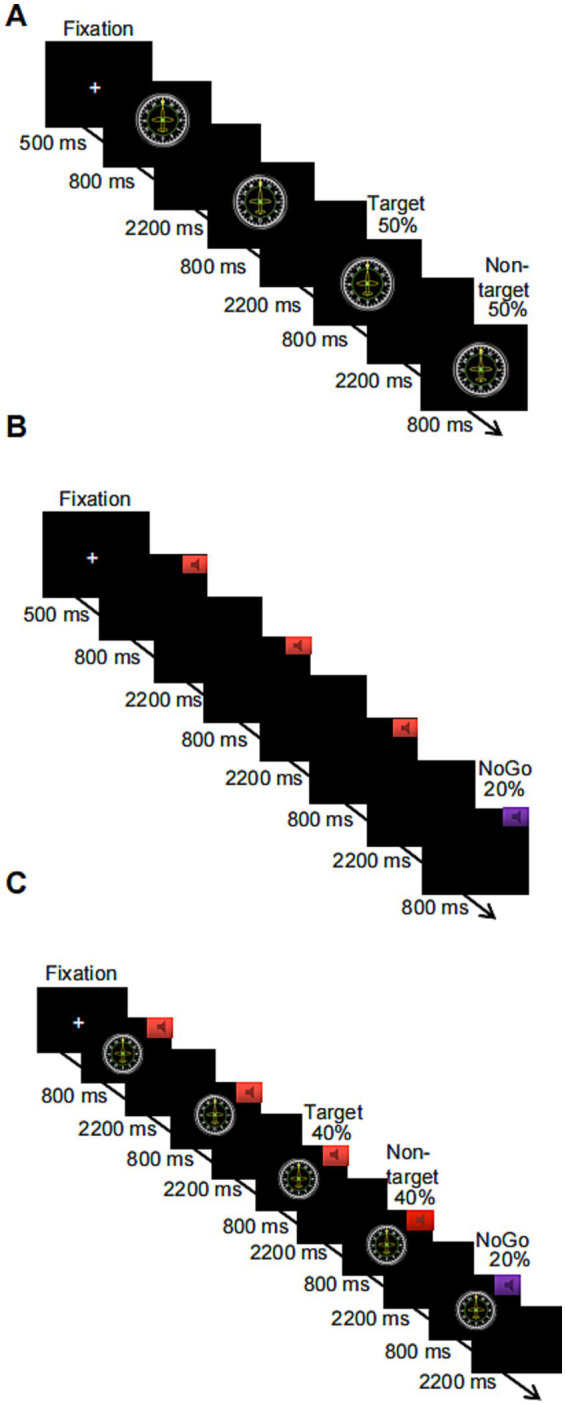
Flowcharts of three levels of picture-based working memory tasks: **(A)** the visual-only 2-back task; **(B)** the auditory-only Go/NoGo task; and **(C)** the combined visual 2-back + auditory Go/NoGo dual task.

The 2-back paradigm was used to assess subject performance on a visual WM task. Before the start of this task, a “+” appeared in the center of the screen for 500 ms to remind subjects to focus on the task. When the “+” disappeared, flight instrument pictures randomly appeared on the computer screen, and the pointer on each flight instrument pointed to a number or a letter (e.g., W, 30, 33, N, 3, 6, E, 12, 15, S, 21, or 24). The picture stimuli lasted 800 ms, and the next picture stimulus was automatically presented after the participant pressed a key or 3,000 ms. The target stimulus and the nontarget stimulus each appeared 50% of the time, and subjects were required to judge whether the number or letter indicated by the pointer of the current flight instrument panel picture was the same as that indicated by the previous pointer according to the instructions. If the stimuli were the same, participants responded by pressing “F” on the keyboard; if the stimuli were different, participants responded by pressing “F” on the keyboard. The task consisted of 1 block with a total of 80 trials. There was a 1-min (20-trial) practice phase before entering the formal experimental task, and feedback was provided on correct or incorrect responses. When the level of accuracy in the practice phase reached 80%, subjects were assumed to understand the task; otherwise, the subject had to repeat the practice phase. The duration of the entire experimental task was approximately 5 min, and the flowchart is shown in Figure 5A. The output was subject response accuracy and reaction time on the target and nontarget stimuli.

##### 3.1.4.2 Auditory-only working memory task

Same as Experiment 1, and the flowchart is shown in Figure 5B.

##### 3.1.4.3 Audiovisual working memory dual task

The 2-back + Go/NoGo combination paradigm was used to assess WM task performance under audiovisual incongruence. Before the start of the task, a “+” appeared in the center of the screen for 500 ms to remind subjects to focus on the task. When the “+” disappeared, any one of the 12 flight instrument panel pictures was randomly presented on the computer screen, and one of the two sound stimuli was randomly played through the headphones. The duration of the sound and picture stimuli was 800 ms, and the next stimulus was automatically presented after the participant pressed a key or 3,000 ms. If the sound stimulus was a high-frequency tone, the subject had to judge whether the number or letter indicated by the pointer in the current flight instrument panel picture was the same as the number or letter indicated by the previous pointer picture. If the stimuli were the same, the subject had to press the “F” key on the keyboard; if the stimuli were not the same, the subject had to press the “J” key on the keyboard. If the acoustic stimulus was a low-frequency tone, subjects were instructed to restrain their response and not press a key, regardless of whether the number or letter indicated by the pointer in the current flight instrument picture was the same or different as the previous number or letter. The trials in which responses were restrained accounted for 20% of the total number of trials in the entire experiment. The task consisted of 1 block with a total of 100 trials. The combined visual 2-back + auditory Go/NoGo dual task had the same number of target trials as the visual-only 2-back task and the auditory-only Go/NoGo task to facilitate subsequent comparisons. Additionally, a 1-min (20-trial) practice phase was provided before participants entered the start of the formal experimental task, with feedback provided on correct or incorrect responses. When the level of accuracy in the practice phase reached 80%, the subject was assumed to understand the task; otherwise, the subject had to repeat the practice phase. The duration of the entire experiment was approximately 6 min, and the flowchart is shown in Figure 5C. The output was subject response accuracy and response time on the target trials and nontarget trials in the 2-back condition as well as the response accuracy and response time on the Go trials and the NoGo trials in the Go/NoGo condition of the dual task.

#### 3.1.5 Data processing

Data were organized and statistically analyzed using Excel 2019 and SPSS 25.0. First, data were screened, and datapoints more than 3 standard deviations from the mean were excluded. The data of all 60 subjects were included in the subsequent statistical analysis. In addition, the behavioral data were analyzed using the statistical software SPSS 25.0, mainly using paired-samples *t* tests to compare the 60 subjects’ accuracy and reaction times between the visual-only 2-back task and the visual 2-back condition of the audiovisual dual task, as well as between the auditory-only Go/NoGo task and the Go/NoGo condition of the audiovisual dual task. Finally, the interference effect of audiovisual interaction on WM task performance was examined in terms of the effect size r_pb_^2^ = t^2^/ (t^2^ + df). The effect size was considered small for 0.010 ≤ r_pb_^2^ < 0.059, medium for 0.059 ≤ r_pb_^2^ < 0.138, and large for r_pb_^2^ ≥ 0.138 (Quan, 2003). For all the above statistical analyses, the significance threshold was set to *α* = 0.05.

### 3.2 Results

#### 3.2.1 Comparison of the auditory-only Go/NoGo task and the combined auditory Go/NoGo task + visual 2-back dual task

The accuracy and reaction time results were compared between the auditory-only Go/NoGo task and the auditory Go/NoGo condition in the audiovisual dual task using the visual 2-back task as the interference stimulus, as shown in Table 5.

**Table 5:** Go/NoGo parameters in single and dual tasks (mean ± standard deviation).

Task type	Memory condition	Accuracy	Reaction time (ms)
Single (auditory-only)	Go/NoGo	0.99 ± 0.04	549.07 ± 140.39
Dual (simultaneous audiovisual presentation)	Go/NoGo+2-back	0.89 ± 0.06	673.55 ± 145.85

**Figure 6:**
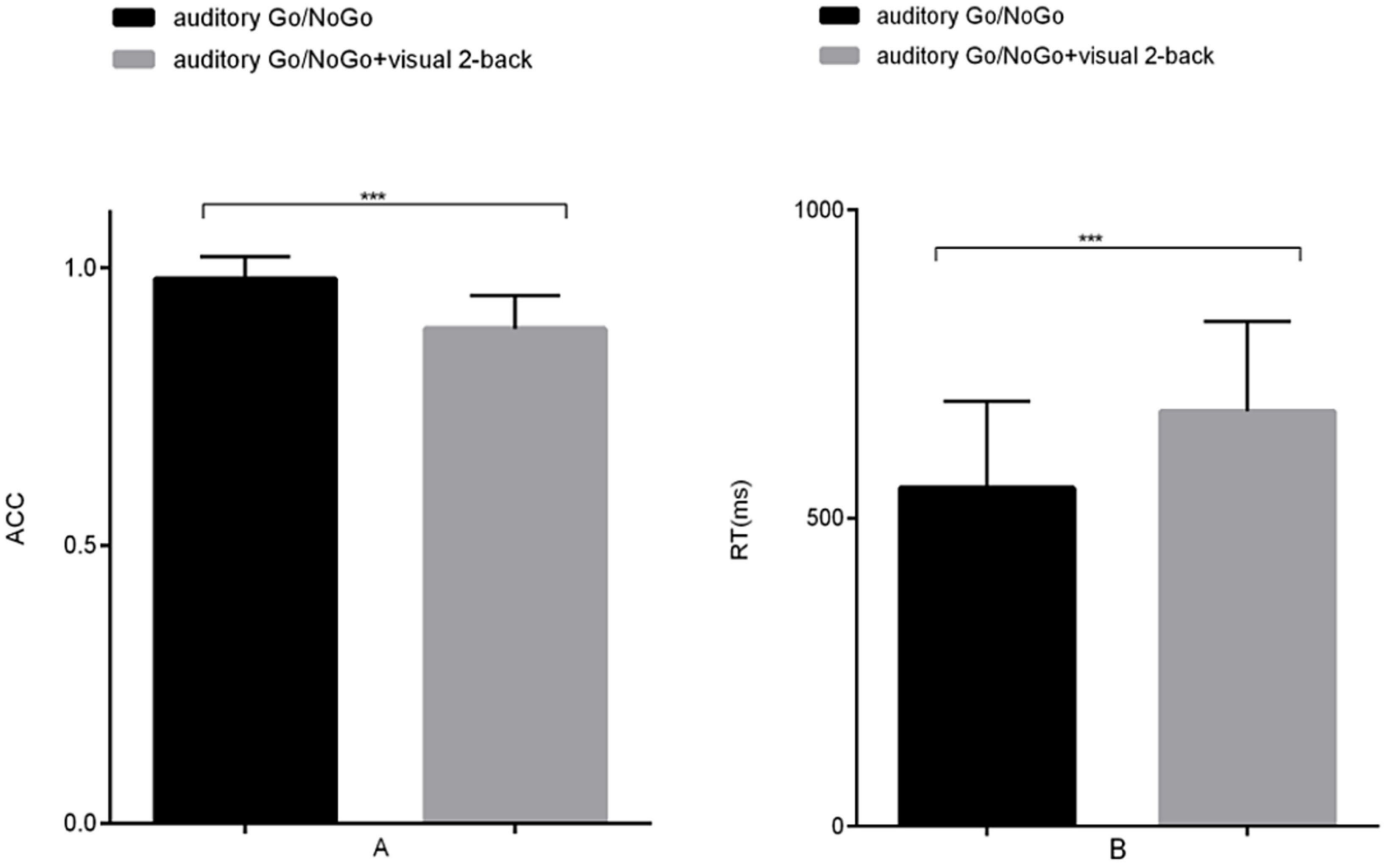
Accuracy and reaction time in the auditory-only Go/NoGo task and audiovisual dual task. **(A)** single-task (auditory) and dual-task (audiovisual) conditions for accuracy (ACC); **(B)** single-task (auditory) and dual-task (audiovisual) conditions for reaction time (RT). Note: ACC denotes response accuracy, RT denotes reaction time, * denotes *p* < 0.05, ** denotes *p* < 0.01, and *** denotes *p* < 0.001.

The accuracy in the auditory Go/NoGo condition of the audiovisual dual task was significantly lower than that in the auditory-only Go/NoGo task [*t* (58) = 2.044, *p* < 0.001, r_pb_^2^ = 0.481] (see Figure 6A). In terms of reaction time, the reaction time in the auditory Go/NoGo condition of the audiovisual dual task was significantly longer than that of the auditory-only Go/NoGo task [*t* (58) = −2.849, *p* < 0.001, r_pb_^2^ = 0.210] (see Figure 6B). Finally, in the combined auditory Go/NoGo + visual 2-back dual task, we calculated the correlation between accuracy and reaction time to determine whether there was a speed-accuracy trade-off. The results showed no significant negative correlation between the two [r (58) = − 0.213, *p* > 0.05].

#### 3.2.2 Comparison of a visual-only 2-Back task with a combined visual 2-back task + auditory Go/NoGo dual task

Using the auditory Go/NoGo task as a distractor stimulus, the accuracy and reaction time results were compared between the visual-only 2-back task and the visual 2-back condition of the audiovisual dual task, as shown in Table 6.

**Table 6:** 2-back parameters in single and dual tasks (mean ± standard deviation).

Task type	Memory condition	Accuracy	Reaction time (ms)
Single (visual-only)	2-back	0.94 ± 0.07	931.05 ± 258.06
Dual (simultaneous audiovisual presentation)	2-back	0.87 ± 0.09	1268.29 ± 285.34.

**Figure 7:**
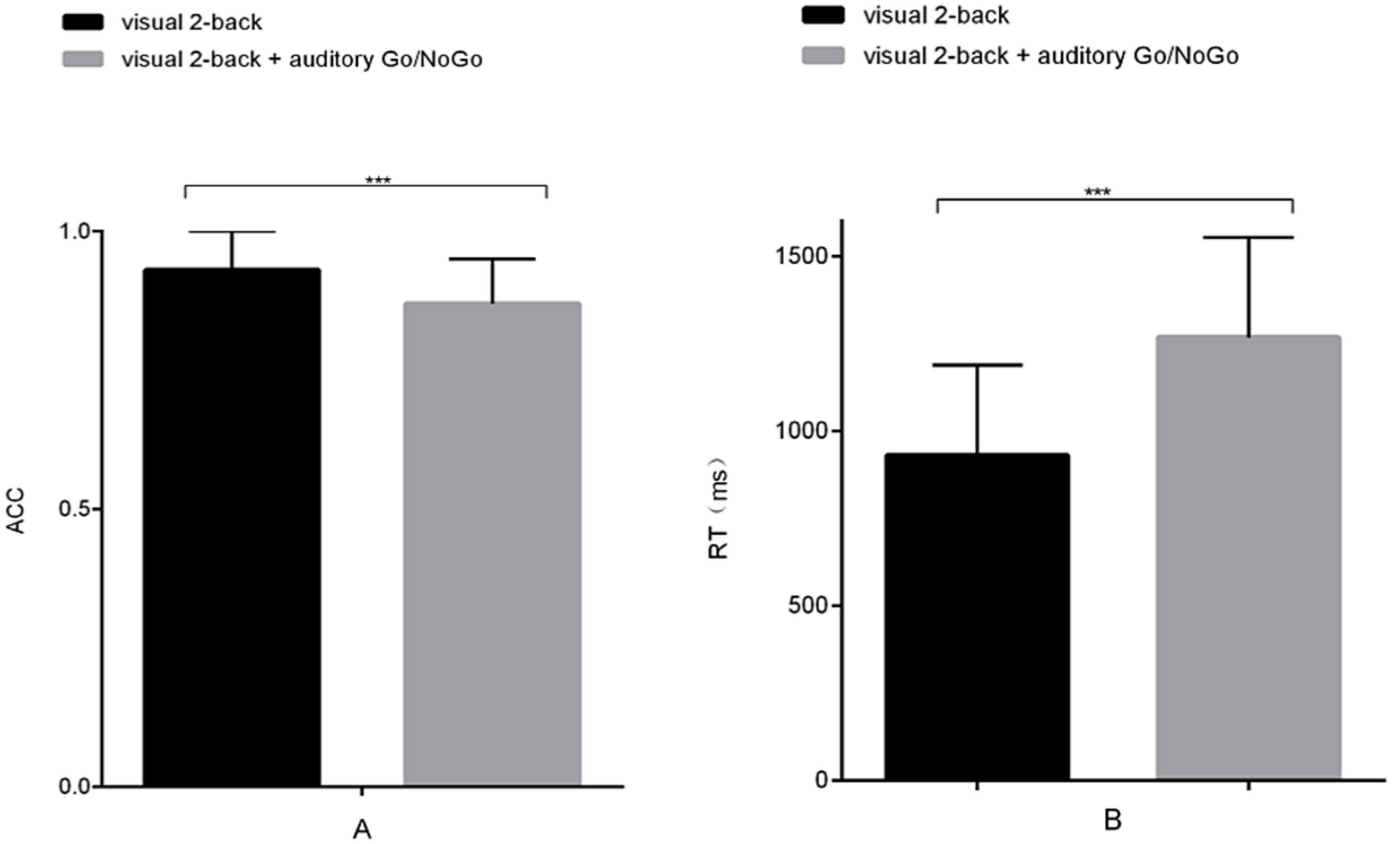
Accuracy and reaction time in the visual-only 2-back task versus the audiovisual dual task. **(A)** single-task (visual) and dual-task (audiovisual) conditions for accuracy (ACC); **(B)** single-task (visual) and dual-task (audiovisual) conditions for reaction time (RT). Note: ACC denotes response accuracy, and RT denotes reaction time. * denotes *p* < 0.05, ** denotes *p* < 0.01, and *** denotes *p* < 0.001.

The accuracy of the visual 2-back condition of the audiovisual dual task was significantly lower than that of the visual-only 2-back task [*t* (58) = 4.142, *p* < 0.001, r_pb_^2^ = 0.225] (see Figure 7A). In terms of reaction time, the reaction time of the visual 2-back condition of the audiovisual dual task was significantly longer than that of the visual-only 2-back task [*t* (58) = −2.258, *p* < 0.001, r_pb_^2^ = 0.664] (see Figure 7B). In addition, in the combined visual 2-back + auditory Go/NoGo dual task, we calculated the correlation between accuracy and reaction time to determine whether there was a speed-accuracy trade-off. The results showed no significant negative correlation between the two [r (58) = −0.186, *p* > 0.05].

### 3.3 Summary

The results of a picture-based audiovisual WM dual task showed that there was bidirectional interference between the visual and auditory channels of information processing for stimuli with audiovisual incongruence. That is, vision interfered with auditory WM task performance, and hearing interfered with visual WM task performance.

## 4 Discussion

The primary objectives of this study were to examine the impact of audiovisual incongruence on WM task performance and to establish a theoretical foundation for WM information processing in such contexts. To attain these research aims, we employed the 2-back task and the Go/NoGo task, which are well-established research paradigms for investigating WM. The 2-back task imposes more complex requirements on WM processing, especially checking for updates, storing and retaining information. This task is presented mainly in the visual modality (Carriedo et al., 2016). The Go/NoGo task is the classic research paradigm for evaluating the ability of WM to inhibit interference. Almost all tasks assessing central executive functions require the involvement of inhibitory functions (Karr et al., 2018). This task is presented mainly in the auditory modality. Therefore, the 2-back task was used to measure subjects’ visual WM task performance, and the Go/NoGo task was used to measure subjects’ auditory WM task performance. Our research findings indicate that, compared with unimodal conditions, audiovisual incongruence impairs WM performance, whether in visual or auditory WM tasks. This reflects an interference effect rather than a facilitative one, which is consistent with our Hypothesis 1.

Specifically, in Experiment 1, when the visual 2-back task was presented as an interfering stimulus in the performance of the auditory-only WM task, the performance in the auditory WM condition in the audiovisual dual task showed a downward trend, reflected in decreased accuracy and longer reaction times. This corresponds to the McGurk effect, in which the visual and auditory stimuli are presented simultaneously but conflict with each other. Neuroimaging evidence suggests that such interference may arise from competitive interactions between the visual and auditory cortices. For instance, functional MRI studies have shown that increased activation in the occipital cortex during visual dominance tasks correlates with suppressed activity in the superior temporal gyrus (STG), a key region for auditory processing (Morís Fernández et al., 2015). When visual and auditory processing compete for limited WM resources, they interfere with each other in terms of information processing, following the theory of resource competition under audiovisual interaction (McGurk and MacDonald, 1976). This cortical competition aligns with the “inverse effectiveness” principle in multisensory integration, where modality-specific cortices exhibit mutual suppression under conflicting inputs (Holmes, 2009). The presence of visual stimuli interferes with the extraction of auditory information (Sandhu and Dyson, 2016), which in turn affects the brain’s ability to make a timely processing response (Evans and Treisman, 2010), leading to longer reaction times and lower accuracy in the auditory WM condition of the audiovisual WM dual task than that in the auditory-only WM task. In addition, Sinnett et al. (2007) found that when audiovisual information was presented simultaneously but the information was incongruent, subjects needed more cognitive resources to suppress the interference of irrelevant visual information relative to auditory information; thus, the processing of information in the visual channel inevitably affected the processing of information in the auditory channel.

Conversely, when the auditory stimulus was presented as an interfering stimulus, there was no difference in accuracy between the visual 2-back condition of the audiovisual dual task and the visual-only 2-back task, while there was a borderline statistically significant difference in reaction time, which was significantly longer in the audiovisual dual task than in the visual-only 2-back task. This asymmetry in cross-modal interference likely reflects differential cortical prioritization mechanisms. Specifically, the ventral visual pathway’s robust bottom-up processing may confer resilience to auditory interference (Wang et al., 2024), while auditory processing in the temporal cortex appears more susceptible to top-down visual modulation facilitated by frontoparietal networks (Braga et al., 2017). We further found a speed-accuracy trade-off, such that subjects sacrificed reaction time to increase accuracy. This finding aligns with Loomis et al. (2012), who documented a similar trade-off in spatial working memory when dual-task demands surpassed cognitive resource capacity. Such strategic reallocation of resources suggests that limited cognitive resources were prioritized for accuracy-critical operations, such as WM updating, which is consistent with the resource competition framework proposed by Wickens et al. (1983). Additionally, considering effect sizes, we found that the presence of auditory interference had only a small effect on visual WM task performance in the audiovisual task, both in terms of reaction time and accuracy (Quan, 2003). This finding aligns with Shipstead et al. (2019), who demonstrated that auditory interference effects, while weaker than visual ones, were not absent.

Similarly, in Experiment 2, when visual stimuli were presented as an interfering stimulus, increases in cognitive load due to visual interference led to a significant decrease in auditory WM task performance in the audiovisual dual task compared with the auditory-only WM task, which was reflected mainly in the substantial decrease in accuracy and prolongation of reaction times, in line with previous research findings. This amplification of interference under high visual load may involve overloaded frontoparietal control networks. When visual processing demands exceed prefrontal capacity, top-down suppression of auditory distractors becomes less effective, exacerbating cross-modal interference (Melara et al., 2021; Molloy et al., 2015). For example, Dehais et al. (2019) found that the recognition of auditory target stimuli was related to the visual cognitive load and that the recognition rate of auditory target stimuli significantly decreased as the visual cognitive load increased, resulting in longer reaction times and significantly lower accuracy in response to target stimuli in the presence of interfering, distracting stimuli in the visual 2-back task (Murphy et al., 2016). Weil et al. (2012) also showed that in audiovisual interactions, auditory stimuli are used as target stimuli and incongruent visual stimuli are used as distractor stimuli, which affects subjects’ brain responses to some extent. The magnitude of this effect depends on the visual cognitive load imposed by the incongruent interfering stimulus, and the interference effect becomes increasingly obvious as the visual cognitive load increases (Khetrapal, 2010).

Conversely, when auditory stimuli were presented as an interfering stimulus, increases in the visual cognitive load in the visual WM condition of the audiovisual dual task led to a significant downward trend in WM performance compared to that in the visual-only WM task with picture stimuli, which was reflected in lower accuracy and longer reaction times in the visual WM refreshing function. This bidirectional interference under high visual load may reflect reduced functional connectivity between the auditory cortex and default mode network (DMN). When visual load depletes prefrontal resources, DMN suppression weakens, allowing auditory distractors to intrude into visual WM maintenance (Chadick and Gazzaley, 2011). This outcome is consistent with the classic sound-induced flash illusion, i.e., when audiovisual stimuli are presented simultaneously but the information is incongruent, resource competition occurs, and the presence of auditory stimuli interferes with the extraction of visual information (Meylan and Murray, 2007). Moreover, the absence of a significant speed and accuracy trade-off under high visual load (picture stimuli) suggests that participants could no longer flexibly balance competing demands. This may reflect global resource depletion, where heightened visual cognitive load (e.g., processing complex instrument panels) consumed attentional reserves, leaving insufficient resources to modulate speed and accuracy (Sinnett et al., 2007; Bigelow and Poremba, 2016). For example, Thelen et al. (2012) similarly found that extreme cognitive load disrupts compensatory strategies, leading to parallel declines in both speed and accuracy—a pattern mirrored in our findings.

These trade-off dynamics provide critical insights into the mechanisms of audiovisual interference. Under low load (Experiment 1), participants leveraged residual resources to prioritize accuracy, whereas under high load (Experiment 2), resource exhaustion forced a “breakdown” of strategic control, resulting in bidirectional interference. This aligns with the load theory of attention (Lavie et al., 2004), where perceptual load determines the capacity to suppress distractors. This is in line with previous studies that have found significant differences between the effects of interfering auditory stimuli and the effects of interfering visual stimuli. One of the biggest differences was that auditory stimuli as interfering stimuli had a greater effect only when the visual cognitive load was high (Tellinghuisen and Nowak, 2003; Alderson et al., 2017). In addition, these findings align with domain-general attentional models and caution against overgeneralizing visual supremacy (Souza et al., 2018). The primary reason for this phenomenon may stem from the flexible, task-dependent nature of attention allocation across sensory modalities (Morey et al., 2013). For instance, Fox et al. (2009) showed that when task demands prioritize relevance, participants can direct greater focus to auditory stimuli over visual inputs in dual-task paradigms. In our study, the structured demands of the visual 2-back task—which necessitates continuous mental updating—inherently consumes more attentional resources. This design feature likely biases interference patterns toward visual dominance rather than reflecting an inherent hierarchical advantage. Consequently, the unidirectional interference observed in Experiment 1 appears more attributable to asymmetrical task constraints than to a fixed modal dominance hierarchy. While visual dominance often manifests in multisensory processing, our work highlights its context-sensitive nature and modulation by cognitive load, aligning with domain-general frameworks of attention (Morey, 2018; Souza et al., 2018) rather than rigid, modality-specific rankings. These findings warrant further investigation into the boundary conditions and adaptive mechanisms underlying cross-modal attention dynamics.

In summary, the outcomes of Experiment 1 demonstrated that under low visual cognitive load, visual information processing exerted stronger cross-modal interference on auditory WM performance than vice versa. This asymmetry aligns with our task-contingent resource competition hypothesis (H2), not fixed perceptual hierarchies. Three lines of evidence support this: (1) mandate-driven resource allocation: the visual 2-back task’s continuous updating demands (monitoring sequential stimuli and comparing them to prior inputs) engaged the dorsal attention network for spatial WM maintenance (Corbetta and Shulman, 2002), reducing resources for suppressing auditory distractors, whereas the auditory Go/NoGo task’s discrete decision-making (respond/withhold) had a smaller resource footprint (Lavie et al., 2004). (2) Modality-neutral competition patterns: despite visual stimuli accounting for 83% of sensory input (Feng and Yang, 2018), neuroimaging shows auditory dominance in temporal prediction tasks (Repp and Penel, 2002), and attention can prioritize auditory inputs when task-relevant (Fox et al., 2009), indicating task architecture—not sensory modality—drives interference. And (3) speed-accuracy trade-offs: the speed-accuracy trade-off (negative correlation between RT and accuracy) under dual-task conditions suggests participants strategically reallocated resources to preserve 2-back accuracy at the expense of slower responses (Loomis et al., 2012), mirroring domain-general WM models where allocation adapts to task priority (Morey, 2018). Thus, our study extends this view by demonstrating that visual interference patterns emerge under conditions of asymmetric cognitive load rather than from intrinsic sensory superiority.

Experiment 2 demonstrated that under high visual cognitive load, there was bidirectional interference between visual and auditory information processing, meaning visual processing disrupted auditory processing and vice versa. The reason for the above results may be that human cognitive processing load is limited by WM, and when cognitive resources are effectively allocated and controlled, cognitive load is negatively correlated with cognitive resource reserve (Wickens et al., 1983), i.e., the lower the cognitive load perceived by an individual, the more adequate the cognitive resource reserve is and the easier it is to suppress irrelevant interfering stimuli. However, during the processing of pictures (which impose a high visual cognitive load), cognitive resources are consumed, and individuals cannot effectively inhibit interfering stimuli, thereby becoming more susceptible to interference (Cheng et al., 2017). Consistent with existing research, compared with performance on the visual-only WM task, performance on the visual WM condition of the audiovisual dual task involved significantly lower accuracy and longer reaction times, with no speed-accuracy trade-off (Bigelow and Poremba, 2016; Thelen et al., 2012). Thus, when auditory interference is present, a significantly stronger interference effect manifests exclusively during high visual cognitive load conditions (e.g., tasks involving complex picture stimuli) compared to low-load conditions (e.g., digit-based tasks). This heightened interference arises from intensified competition for limited working memory resources, as posited by Hypothesis 3. Specifically, the elaborate processing demands of visual tasks requiring object recognition and spatial analysis (characteristic of picture stimuli) leave fewer residual resources to counteract auditory distraction. In contrast, low-load digit tasks—which involve minimal visual feature analysis—retain sufficient attentional capacity to mitigate cross-modal interference. These findings provide robust empirical support for our hypothesis that the magnitude of cross-modal interference is directly modulated by the cognitive demands imposed by the primary visual task, thereby underscoring the dynamic interplay between task complexity and attentional resource allocation (Colombo and Graziano, 1994; Shipstead et al., 2019).

Notably, our findings do not negate auditory dominance in contexts like the sound-induced flash illusion (Shams et al., 2000) or flexible attention shifts reported by Fox et al. (2009). Rather, they highlight that interference patterns depend on task demands and resource availability. However, there are some limitations in this study that need to be addressed and improved in future research. The first and most critical limitation is that while the present study explored the effects of audiovisual incongruence on WM task performance, we did not increase auditory task difficulty and only increased visual task difficulty. As posited by Posner et al. (1976), future studies must systematically vary auditory task difficulty to dissociate modality-specific and domain-general effects. Second, the entire experiment was behavioral in nature and lacked direct electrophysiological evidence. In future studies, more advanced instrumentation and techniques, such as electroencephalography (EEG), near-infrared spectroscopy, and functional magnetic resonance imaging, should be employed to conduct an in-depth exploration of information processing between the visual and auditory channels during audiovisual incongruence.

## 5 Conclusion

Our findings reveal cognitive load as a critical moderator of cross-modal interference patterns, rather than visual dominance per se. Under low load conditions, task constraints drove asymmetric interference, with audiovisual incongruence disproportionately affecting performance in one direction—for instance, visual distraction impaired auditory processing more than auditory distraction impaired visual processing. Conversely, under high load conditions, global resource depletion facilitated bidirectional disruption, wherein both visual and auditory distractors impaired WM performance. This challenges rigid sensory hierarchy models and aligns with domain-general attention frameworks, which propose that attentional resources are dynamically allocated based on task demands rather than fixed sensory priorities. Future research must disentangle modality-specific and shared-resource mechanisms through balanced load manipulations, systematically varying both visual and auditory cognitive loads to elucidate the boundary conditions of cross-modal interference and refine theoretical models of multisensory processing.

## Data Availability

The original contributions presented in the study are included in the article/supplementary material, further inquiries can be directed to the corresponding authors.
